# Polycarbosilane/Divinylbenzene-Modified Magnesium Hydroxide to Enhance the Flame Retardancy of Ethylene–Vinyl Acetate Copolymer

**DOI:** 10.3390/polym15224440

**Published:** 2023-11-17

**Authors:** Siyuan Li, Chunfeng Wang, Guodong Wang, Yongliang Wang, Zhidong Han

**Affiliations:** 1 School of Materials Science and Chemical Engineering, Harbin University of Science and Technology, Harbin 150040, China; lisiyuan@stu.hrbust.edu.cn (S.L.); 17865353256@163.com (G.W.); yongliangwang@hrbust.edu.cn (Y.W.); zhidong.han@hrbust.edu.cn (Z.H.); 2 Key Laboratory of Engineering Dielectrics and Its Application, Ministry of Education, Harbin University of Science and Technology, Harbin 150080, China

**Keywords:** ethylene–vinyl acetate copolymer, magnesium hydroxide, polycarbosilane, divinylbenzene, cone calorimeter, total smoke production

## Abstract

The thermal decomposition product of magnesium hydroxide (MH) is magnesium oxide (MgO), which serves as the foundational material for fireproof layer construction in the condensed phase. However, the weak interaction force between particles of MgO generated by thermal decomposition leads to the insufficient strength and poor adhesion ability of the fireproof layer. The fireproof layer was easily damaged and detached in this study, resulting in the low flame-retardant efficiency of MH. In this work, polycarbosilane (PCS) and divinyl benzene (DVB) were used to modify MH, and EVA/MH/PCS/DVB composites were made via melt blending. The flame-retardant properties of EVA/MH/PCS/DVB were evaluated using the limiting oxygen index (LOI), vertical combustion (UL-94), and a cone calorimeter (CONE). The thermal stability of the composites and flame retardants was analyzed using a thermogravimetric analyzer. The char layer structure was observed and analyzed using scanning electron microscopy (SEM) and X-ray photoelectron spectroscopy (XPS), respectively. The results indicate that the LOI of the EVA/MH/PCS/DVB with 50 wt.% flame retardants in total was as high as 65.1, which increased by 160% in comparison with EVA/MH. Furthermore, the total smoke production (TSP) of the EVA/MH/PCS/DVB composite decreased by 22.7% compared to EVA/MH/PCS; the thermal stability of the MH/PCS/DVB and EVA/MH/PCS/DVB improved to some extent; and the compact residual char after the combustion of EVA/MH/PCS/DVB had fewer cracks due to the adhesive effect induced by PCS/DVB.

## 1. Introduction

Ethylene–vinyl acetate copolymer (EVA) is a thermoplastic resin made from ethylene and vinyl acetate [1] that has lower crystallinity due to the vinyl acetate monomer (VA) in its molecular chains. EVA is widely used in the wire and cable industry for its excellent weather resistance, elasticity, and electrical insulation [2,3,4]. However, it is essential to note that EVA has a lower limiting oxygen index (LOI) of only 17.9, making it highly flammable. Moreover, the combustion process of EVA produces toxic gases such as CO [5]. Therefore, it is necessary to improve the flame retardancy of EVA; adding flame retardants can effectively reduce the flammability of EVA [6]. In terms of green, environmentally friendly, and sustainable development, halogen-free, non-toxic, and low-smoke flame retardants have become a research hotspot in the field of flame retardancy in recent years [7].

The preparation and application of the inorganic flame retardants represented by MH and alumina trihydrate (ATH) [8] have no impact on the environment compared with halogen-based and phosphorous-based flame retardants, which aligns with the current research direction of flame retardants [9]. The thermal decomposition of MH mainly occurs at 350 °C~400 °C, which can suppress the surface temperature increases in the composites. The generated water vapor can dilute the concentration of combustible gas, and the generated magnesium oxide covering the surfaces of the composites could hinder the heat and mass transfer between the gas phase and the condensed phase [10]. In consideration of the flame-retardant mechanism of MH, it is necessary to add certain amounts of flame retardants to achieve the desired flame-retardant effect. However, an excessive addition will deteriorate the mechanical properties of the composites. The problem of attaining high-efficiency, halogen-free, and low-smoke EVA composites still urgently needs to be solved [11].

To improve the flame-retardant effect of MH, researchers have conducted extensive research, including the preparation of nanoscale MH [12,13]; the modification of MH, such as using melamine cyanurate [14], cyano trimethylene triphosphate [15], triethoxysilane, and polymethyl vinyl silicone rubber [16]; and the use of multi-element synergistic flame-retardants such as hexa (4-boric acid phenoxy) cyclophosphamide [17], cellulose nanofiber [18], expandable graphite [19], carbon black [20], silicone rubber [21], zinc borate [22], montmorillonite [23], silicon dioxide [24], graphene [25], carbon nanotubes [26], and hollow glass microspheres [27]. Based on the flame-retardant mechanism of MH and the self-sustaining combustion cycle theory, the carbon layer formed by the accumulation of magnesium oxide particles has a crucial impact on the combustion behavior of materials; therefore, the strength and density of the carbon layer are critical factors in the flame-retardant mechanism of the condensed phase [28,29].

Polycarbosilane is an ideal precursor for preparing silicon carbide ceramic materials. The molecular structure of PCS is mainly composed of three primary substructures, namely, the ideal Si-C linear (L) units, six-membered Si-C monocyclic (SR) units, and Si-C condensed ring (CR) units [30]. Based on previous research by our group, we have reported the significant flame-retardant effect of PCS in collaboration with MH in polyethylene [31,32] and ethylene–vinyl acetate [11] copolymers. The physical barrier effect of the carbon layer had an outstanding improvement. However, the smoke production increased due to the small-molecule silane gas release generated by PCS degradation; for example, the smoke production of EVA/MH composites with 2 wt.% PCS increased by 45%, and adding 3% increased smoke production by 59%. To improve the ceramic yield of PCS, divinyl benzene (DVB) is usually used as a cross-linking agent [33]. In view of this, the modification of MH with PCS and DVB (MH/PCS/DVB) was prepared, and the flame retardancy and combustion behaviors of the flame-retardant EVA composites were studied. It was expected that PE/MH/PCS/DVB composites would enhance the flame-retardant properties while effectively inhibiting smoke release, thus improving the fire safety of the composites and providing an effective means of preparing high-performance composites, especially for wires and cables.

## 2. Materials and Methods

### 2.1. Materials

Commercial ethylene–vinyl acetate copolymer (EVA, 7470m, 26 wt.% vinyl acetate, melt flow index: 4.0 g/10 min) was supplied by Formosa Plastics Co., Ltd., Ningbo, China. Magnesium hydroxide (MH, 5-C) was obtained from Dandong Songyuan Chemicals Co., Ltd., Dandong, China. Polycarbosilane (PCS) with a relative molecular mass of 1320 and 99% purity was sourced from Suzhou Sailifei Ceramic Fiber Co., Ltd., Suzhou, China. Divinylbenzene (DVB) with a 55% mixture of isomers was purchased from Shanghai Mcllean Biochemical Co., Ltd., Shanghai, China.

### 2.2. PCS Cross-Linking Degree Test

PCS and DVB were dissolved in a xylene solution in specific proportions, and the resulting solution was placed in a drying oven at 120 °C for 4 h to obtain solid samples. These solid samples were then wrapped with filter paper and metal mesh before being submerged in a boiling xylene reagent for reflux extraction for 30 min. During the reflux extraction process, PCS and DVB without cross-linking were dissolved in a xylene solution. The mass of the samples before and after extraction was accurately measured to determine the gel content, which was expressed as the percentage of residual mass after extraction compared to the initial sample mass. The cross-linking degree was characterized based on the gel content.

### 2.3. Preparation of MH/PCS/DVB

**Figure 1:**
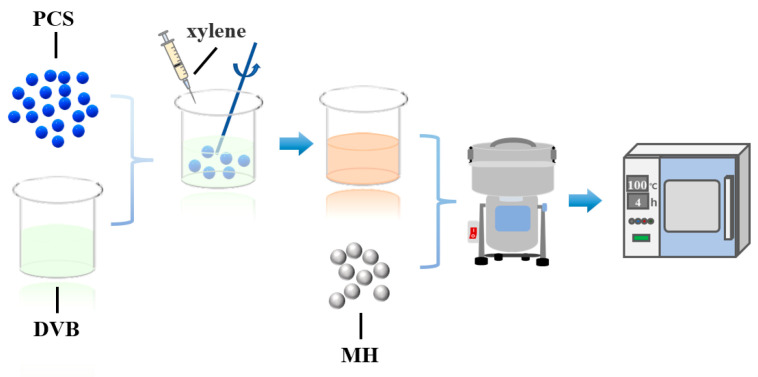
Synthesis process of MH/PCS/DVB.

PCS and DVB were weighed with a mass ratio of 2:1 and dissolved in a xylene solvent to obtain a PCS/DVB solution. The prepared PCS/DVB solution was added drop by drop to MH and mixed at a high speed of 25,000 rpm/min for 2 min using a multi-functional grinder. After thorough mixing, the mixture was dried in an oven at 100 °C for 4 h. The same method was used in the preparation of MH/PCS. The MH/PCS/DVB preparation process is illustrated in Figure 1.

### 2.4. Preparation of Composites

The composites were prepared using a torque rheometer (Harbin Hapro Electric Technology Co., Ltd., Herbin, China). The flame retardant and EVA were melt-mixed at 130 °C for 5 min using the compositions shown in Table 1. Subsequently, the mixtures were pressed and molded using a flatbed vulcanizing machine (JB-25, Shanghai Jiubin Instrument Co., Ltd., Shanghai, China) at 130 °C and 10 MPa for 3 min, with the samples measuring 100 mm × 100 mm × 3 mm. It is important to note that EVA/MH/PCS/DVB was compared with EVA/MH/PCS+DVB, where DVB was added during the melt blending process.

### 2.5. Characterization

The water contact angle test used the SZ-CAMA1 equipment from the Shanghai Xuanzhun Instrument Company (Shanghai, China). Before the test, the tablet press was used to maintain the pressure at 10 MPa for 5 min and press the sample into the shape of a round cake. The sample was then gently placed on the equipment to test its contact angle with 4 μL water droplets.

X-ray diffraction (XRD) was conducted on a diffractometer (X’PeRT PRO, PANalytical, Almelo, The Netherlands) with Cu Kα (1.5406 A), a test resolution of 4 cm^−1^, and a scanning range of 5–80°.

Fourier transform infrared spectroscopy (FTIR) was performed using the NICOLET iS10 infrared spectrometer manufactured by Thermo Fisher Scientific, Waltham, MA, USA. The spectral range was 4000~400 cm^−1^, and the highest resolution was 0.4 cm^−1^. The test resolution was 4 cm^−1^ with 16 scans. The solution of the test was 4 cm^−1^.

The limiting oxygen index (LOI) test was performed using an oxygen index meter (HC-2, Jiangning Analysis Instrument Company, Nanjing, China) with samples measuring 120 mm × 6.5 mm × 3 mm according to the ASTMD 2863-97 standard [34].

A UL-94 vertical burning test was carried out using a CZF-Ⅲ vertical burning tester (Jiangning Analysis and Instrument Company) with samples measuring 127 mm × 127 mm × 3 mm according to the ASTMD 3801 standard [35].

The combustion behaviors were evaluated according to ISO 5660-1 [36] on a CONE calorimeter (6810, Suzhou Yangyi Voucu Testing Technology Co., Ltd., Suzhou, China) with 35 kW/m^2^ heat flux. Various parameters such as the heat release rate (HRR), total heat release (THR), total smoke release (TSP), and mass loss (MASS) were obtained via the CONE test.

A TGA 4000, produced by the PerkinElmer Company of the United States (Waltham, MA, USA), was used for a thermogravimetric analysis. The test atmosphere was nitrogen, the heating rate was 10 °C/min, and the test temperature range was 50–800 °C.

The surface elemental compositions of the char residues were analyzed via X-ray photoelectron spectroscopy (XPS) on a PHI Quantera-II SXM (Ulvac-PHI, Chigasaki, Japan).

Scanning electron microscopy was carried out using the FEI200 scanning electron microscope of the FEI Company, Eindhoven, the Netherlands and the EDAXGenesis2000 energy spectrometer component.

## 3. Results and Discussion

### 3.1. Cross-Linking Degree of PCS/DVB

**Figure 2:**
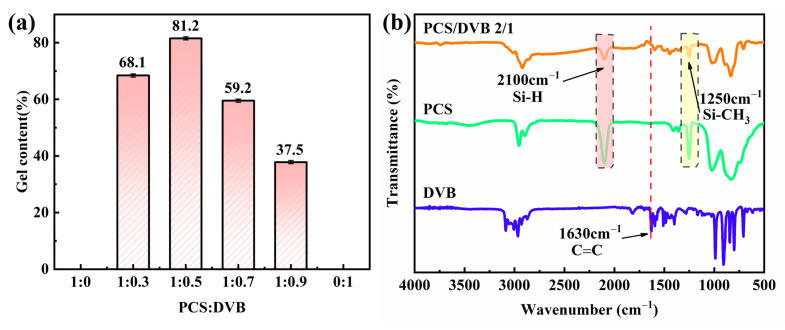
(**a**) The gel content of PCS/DVB after 4 h at 140 °C. (**b**) The FTIR spectra of DVB, PCS, and PCS/DVB.

The Si-H bond exhibits the weaker bond energy (303.8 KJ/mol) in the structure of PCS. The cross-linking behavior of PCS can be conducted by vinyl groups in DVB by self-cross-linking through dehydrogenation. DVB itself can also undergo self-polymerization [37]. The gel content of PCS/DVB is shown in Figure 2a. No gel was left after 4 h at 120 °C for PCS and DVB, which indicates no cross-linking at the temperature of 120 °C after 4 h. It is worth noting that DVB quickly evaporates and depletes at this temperature, while the gel content of the homogeneous mixture of PCS and DVB was outstanding, suggesting a cross-linking reaction between PCS and DVB. The gel content was as high as 81.2% at the mass ratio of PCS to DVB of 1:0.5. In comparison, the gel contents of the other three were much lower than 81.2%, indicating that when the DVB content is low, the cross-linking between PCS molecular chains is linked by the DVB, while at the higher DVB content, there is a greater chance of DVB molecules meeting, leading to self-cross-linking and depletion, which result in inefficient cross-linking of PCS by DVB. Therefore, in this work, the PCS-to-DVB mass ratio was 1:0.5 for the subsequent tests.

The FTIR spectra of DVB, PCS, and PCS/DVB are shown in Figure 2b, and detailed information is given in Table 2. For DVB, the absorption bands at 3100~3000 cm^−1^ and 3000~2850 cm^−1^ are attributed to the stretching of C-H in benzene and the stretching of C-H in methylene, respectively. The absorption bands at 910 cm^−1^ ~700 cm^−1^ are attributed to the out-of-plane bending of C-H in benzene, while the absorption peak at 989 is the out-of-plane bending of C-H in vinyl. For PCS, there are three absorption peaks at 2100 cm^−1^, 1250 cm^−1^, and 830 cm^−1^, which are attributed to the stretching of Si-H, the deformation of Si-CH_3,_ and the stretching of Si-C in PCS, respectively.

In comparison with PCS and DVB, the FTIR curve of PCS/DVB contains both PCS and DVB absorption behaviors, such as the absorption peaks at 3015 cm^−1^, 1595 cm^−1^, 1500 cm^−1^, 1445 cm^−1^, 1410 cm^−1^, and 1359 cm^−1^ resulting from DVB, while the absorption peaks at 2100 cm^−1^, 2150 cm^−1^, and 830 cm^−1^ result from PCS. It is worth noting that the C=C absorption peak at 1630 cm^-1^ disappears, the absorption peak intensity of Si-H at 2100 cm^−1^ and Si-CH_3_ at 1250 cm^−1^ decreases, and the Si-H group is depleted, which indicates cross-linking behavior between PCS and DVB.

### 3.2. Properties of MH/PCS/DVB

**Figure 3:**
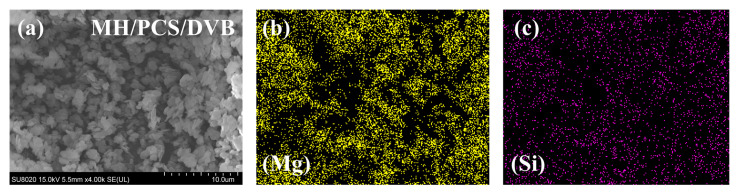
The morphology of MH/PCS/DVB (**a**) and element distributions of Mg (**b**) and Si (**c**).

Tests of the microstructure, surface polarity, X-ray diffraction behavior, and thermodegradation of MH/PCS/DVB were conducted to reveal the properties of MH/PCS/DVB. The microstructure of MH/PCS/DVB and the distribution of Mg and Si are shown in Figure 3. MH/PCS/DVB maintains the hexagonal platelet-shaped and homogeneous distribution of Mg and Si on the surface, which indicates good surface coating of MH by PCS.

**Figure 4:**
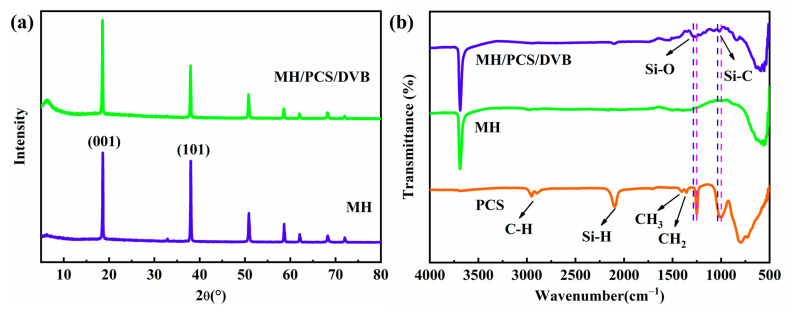
(**a**) XRD patterns and (**b**) FTIR spectra of MH and MH/PCS/DVB.

The X-ray diffraction pattern (a) and the FTIR absorption spectrum (b) of MH/PCS/DVB are shown in Figure 4. In comparison with MH, no new diffraction peak emerged in the diffraction pattern of MH/PCS/DVB (Figure 4a), but there was a noticeable change in the diffraction peak intensity of the (101) lattice plane. The lattice plane (001) of MH exhibited weak polarity, whereas the (101) lattice plane was composed of Mg^2+^ with solid polarity. Thus, the polarity could be analyzed using the ratio of the diffraction peak intensities of the (001) and (101) lattice planes [10]. The I_001_/I_101_ ratios of MH and MH/PCS/DVB were 0.65 and 0.94, suggesting that the surface polarity of MH/PCS/DVB decreased.

Compared with MH and PCS, the Si-C bond of MH/CPS/DVB shifted from 795 cm^−1^ to 833 cm^−1^, while the Si-O bond shifted from 1011 cm^−1^ to 1108 cm^−1^. This indicates a strong interaction between PCS and MH, which is probably due to the interaction of Si-O with Mg^2+^ in the (101) lattice plane as well as the interaction of Si-H with the hydroxyl group in MH, which results in a weakened diffraction pattern of the (101) lattice plane and restricts the movement of the Si-C and Si-O bonds.

**Figure 5:**
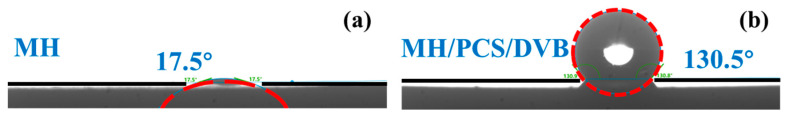
Digital images of the water contact angles of MH (**a**) and MH/PCS/DVB (**b**).

Digital images of the water contact angles of MH and MH/PCS/DVB are shown in Figure 5. The water contact angle of MH is 17.5°, while the water contact angle of MH/PCS/DVB is 130.5°. This implies a transformation of MH powder from hydrophilic to hydrophobic and that the PCS/DVB effectively covered the MH.

**Figure 6:**
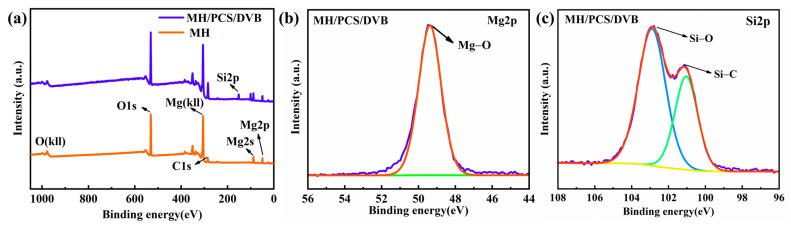
The total XPS spectra (**a**) of MH and MH/PCS/DVB. High-resolution XPS spectra of Mg2p (**b**) and Si2p (**c**).

XPS was conducted to reveal the element bonding state of MH/PCS/DVB. The full XPS spectra of MH and MH/PCS/DVB and the atomic contents of elements are shown in Figure 6 and Table 3, respectively. The binding peak of the Si element is found in the full XPS spectrum of MH/PCS/DVB (Figure 6a), and the binding state of Si2p is revealed in Figure 6c. The binding energies of Si2p at 102.9 eV and 101.1 eV belong to Si-O and S-C, which are dominant in PCS. In addition, the element content on the surface of MH/PCS/DVB was different from MH. The Si content of MH/PCS/DVB increased to 11.27%; the Mg content decreased from 22.78% to 7.76%; and the C content increased from 18.2% to 39.99%. The former atomic content of the C element in MH (18.20%) was due to the contamination of C during the XPS test, while the latter in MH/PCS/DVB (39.99%) was due to PCS/DVB, which indicates that the MH was coated by PCS/DVB.

**Figure 7:**
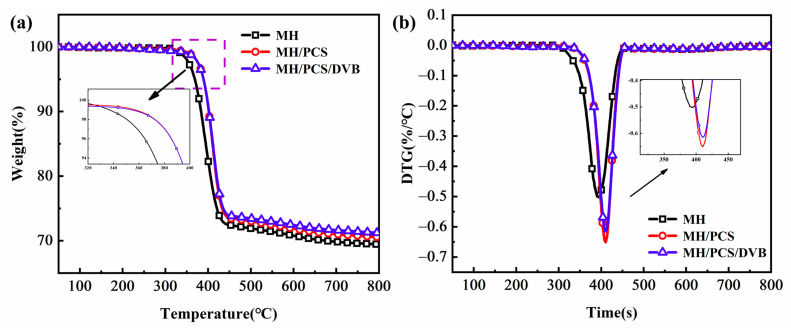
TG (**a**) and DTG (**b**) curves of flame retardants.

The TG and DTG curves of MH/PCS/DVB and their specific parameters are shown in Figure 7 and Table 4, respectively. It can be seen that the thermal degradation curves of MH/PCS and MH/PCS/DVB shifted to a higher temperature in comparison with MH. The initial thermal degradation temperature (T_5_) of MH/PCS and MH/PCS/DVB increased by 20 °C from 369 °C to 389 °C, indicating a significant improvement in the thermal stability of MH after modification. The T_5_ of MH/PCS/DVB was the same as that of MH/PCS, while the T_10_ and T_max_ were higher than those of MH/PCS. The gap increased gradually with the increase in temperature (Table 4). The Rmax of MH/PCS/DVB was −0.73 °C/min weaker than that of MH/PCS (−0.91 °C/min). In addition, the residue at 600 °C of MH/PCS/DVB was 72.4% higher than that of MH/PCS by 0.5 points, which indicates that the thermostability of modified MH can be improved by surface modification with PCS and PCS/DVB and that the mass loss of MH is further inhibited by PCS/DVB. Such a discernible difference in MH modified by PCS and PCS/DVB is due to the cross-linking degree of PCS. The latter is much higher than the former, as evidenced in Figure 2. The results indicate that the cross-linking treatment of PCS with DVB is beneficial for improving the thermal degradation behavior of MH.

### 3.3. The Microstructure, Flame-Retardant Properties, and Mechanical Properties of EVA Composites

**Figure 8:**
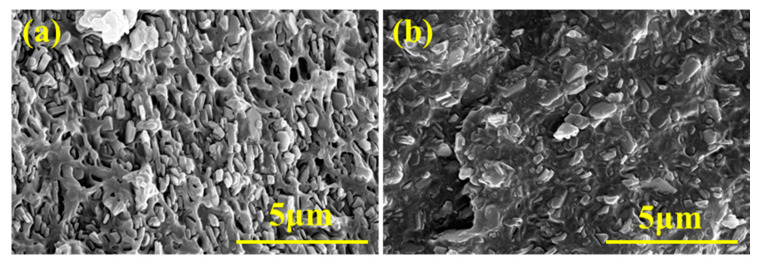
SEM micrographs of EVA/MH (**a**) and EVA/MH/PCS/DVB (**b**).

The microstructures of EVA/MH and EVA/MH/PCS/DVB composites are shown in Figure 8. It can be seen that the MH particle is partially exposed and partially embedded in the EVA matrix (Figure 8a). A noticeable gap exists between MH particles and the EVA matrix, suggesting poor compatibility of MH within the EVA matrix. The MH particle is well covered by EVA and no gap is observed between MH/PCS/DVB and the EVA matrix (Figure 8b), indicating a substantial enhancement of the compatibility between MH and the EVA resin following the PCS and DVB coating modification.

**Figure 9:**
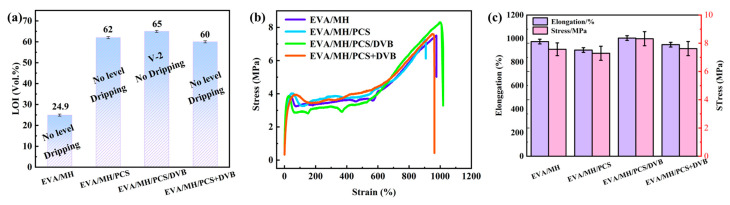
Flame retardancy of composites (**a**), stress–strain curves of EVA composites (**b**), and histogram of EVA composites (**c**).

The flame retardancy of EVA/MH/PCS/DVB is given in Figure 9a. The limiting oxygen index (LOI) of EVA/MH with 50 wt.% MH content was only 24.9, while the LOI values of EVA/MH/PCS and EVA/MH/PCS/DVB were high at 62.0 and 65.1, with increases of 149% and 161% in comparison with EVA/MH, respectively. It is interesting that the vertical burning behavior of EVA/MH/PCS was not improved, while the UL-94 of EVA/MH/PCS/DVB could reach a V-2 rate and the LOI value further increased by 12 percentage points compared with EVA/MH/PCS. Significantly, the flame retardancy of EVA/MH/PCS/DVB was further improved based on the high LOI of EVA/MH/PCS. In contrast, the flame retardancy of EVA/MH/PCS+DVB was decreased, which means the PCS cross-linked by DVB had a more significant synergistic effect with MH than with PCS itself.

The mechanical properties of the EVA composites were characterized via tensile tests. The tensile strength and elongation at break data of the EVA composites are shown in Figure 9b,c. The tensile strength and elongation at break of EVA/MH/PCS were decreased compared to EVA/MH; on the other hand, the cross-linking of PDC/DVB improved both mechanical properties of the EVA composites. The results show that the introduction of PCS/DVB can improve the mechanical properties of EVA composites, and the resulting EVA composites can meet the wire and cable sheathing requirements.

**Figure 10:**
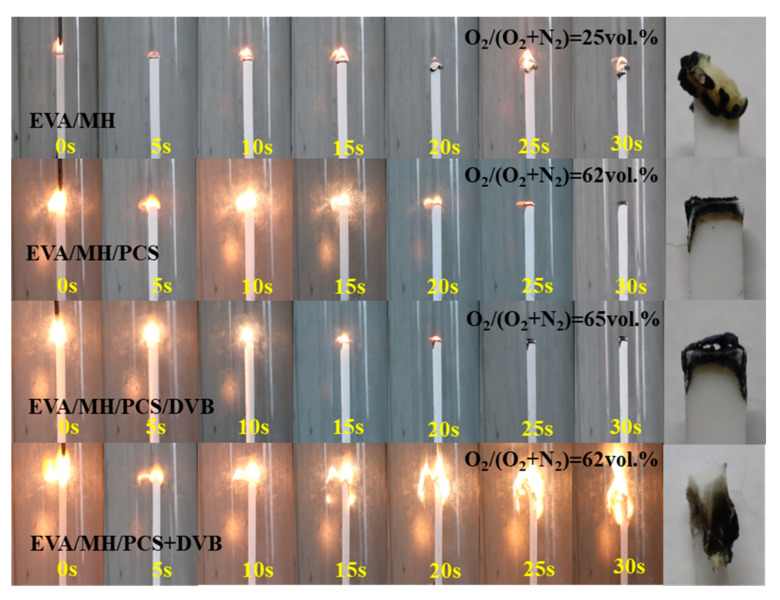
Snap images of the combustion state and the residue after the LOI test.

The combustion states of EVA composites at different combustion times during the LOI test are shown in Figure 10. The combustion of the EVA/MH composite was slow and showed a mitigatory flame during the test. The char on the top of the composite was in the cycle of accumulation and shedding. While the combustion of the EVA/MH composites with PCS was drastic and showed a roaring flame, the char was firmly fixed on the top of the composites once formed, and the flame was effectively restricted and died out. However, the combustion of EVA/MH/PCS+DVB was intensified compared with EVA/MH/PCS/DVB and EVA/MH/PCS, which indicates an enhanced synergistic effect between MH and PCS cross-linked by DVB.

### 3.4. Combustion Behaviors of EVA Composites

**Figure 11:**
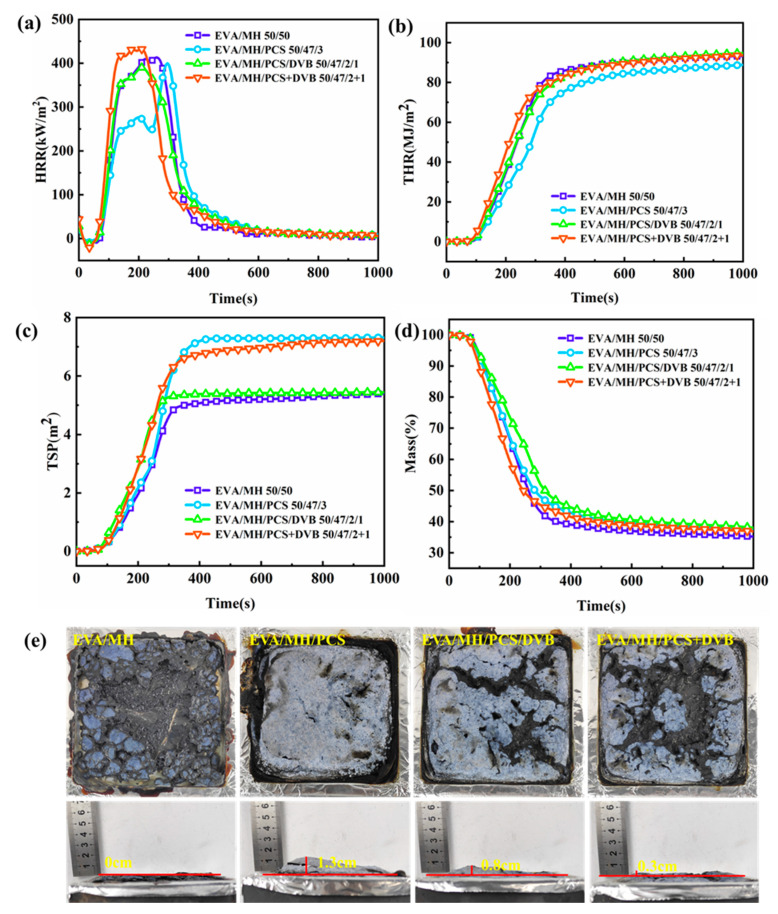
The combustion behaviors of EVA composites: (**a**) HRR, (**b**) THR, (**c**) TSP, (**d**) mass, and (**e**) digital photos of residual char after the CONE test.

The combustion behavior of EVA/MH/PCS/DVB is illustrated in Figure 11, and the combustion parameters are presented in Table 5. The HRR curve of EVA/MH/PCS was different from EVA/MH. The HRR of EVA/MH/PCS was effectively suppressed, especially before 250 s compared with EVA/MH (Figure 11a), and the THR_6min_ of EVA/MH/PCS (84 MJ/m^2^) was higher than that of EVA/MH (90 MJ/m^2^) by 6 percentage points (Figure 11b and Table 5), while the TSP of EVA/MH/PCS (7.3 m^2^) was increased by 40%, which was closely related to the formation of the condensed residue. The tarry and bubbly surface of the residue formed during the combustion of EVA/MH. The bubble cracked back and forth, and thus no intumescent phenomenon was found for the low-strength surface char of the residue (Figure 11e). For EVA/MH/PCS, the migration of PCS to the surface during the combustion helped enhance the strength of the surface char [11], leading to a condensed and cohesive surface char as well as obvious intumescent behavior, which were effective in reducing the HRR, but the migration of PCS also releases more small molecular degradation products to the gas phase, which can increase the TSP.

For the EVA/MH/PCS/DVB composite, the HRR was similar to EVA/MH, which means MH/PCS/DVB had little effect in improving the heat release behavior of the EVA composites compared with MH/PCS, but it was more effective than MH/PCS+DVB (Figure 11a). It is worth noting that the TSP was decreased by 26% compared with EVA/MH/PCS and 21% compared with EVA/MH/PCS+DVB (Figure 11c). In addition, the residue was higher than EVA/MH/PCS by 1.8 percentage points and higher than EVA/MH/PCS+DVB by 1 percentage point (Figure 11d). Such differences among EVA/MH/PCS, EVA/MH/PCS/DVB, and EVA/MH/PCS+DVB are related to the PCS contents and states in the composites and the char residue formation. PCS migrates significantly during combustion. For the EVA/MH/PCS/DVB composite, the continuity of the surface char was inferior to EVA/MH/PCS but obviously superior to EVA/MH/PCS+DVB (Figure 11e), indicating that the 2 wt.% PCS in the composite was not enough to form a continuous surface char, but the continuity could be improved when the PCS was cross-linked by DVB, which led to the apparent differences in HRR and TSP.

According to the combustion parameters of the composites, the fire performance index (FPI), fire hazard growth index (FGI), heat release index (THRI), and smoke emission index (TSPI) were calculated to further evaluate the fire safety of the composites [38]. The fire safety index values of the composites are shown in Table 5. Compared with EVA/MH, the FGI of EVA/MH/PCS decreased by 7.6% and the TSPI increased by 29%. The FGI of EVA/MH/PCS/DVB increased by 11%, while the TSPI had little change. In an actual fire scenario, especially in an unventilated environment, smoke usually causes more death than fire.

### 3.5. Thermal Degradation of EVA/MH/PCS Composites

**Figure 12:**
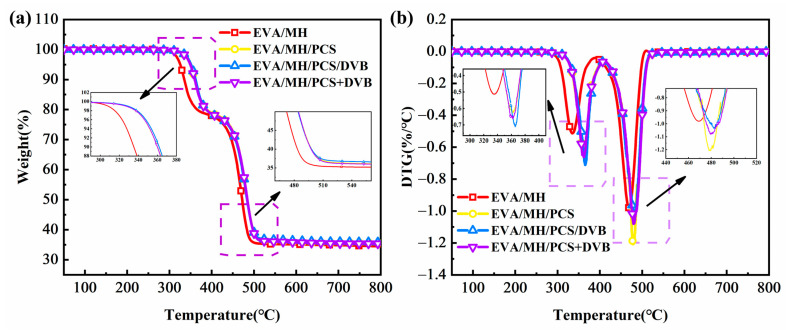
TG (**a**) and DTG (**b**) curves of the composites.

The thermal degradation behavior of the EVA composites is given in Figure 12. There are two stages in the weight loss of EVA composites [39]. The first weight loss stage, at 300–380 °C, is primarily due to the removal of ester groups in the EVA matrix and the release of partial crystalline water in MH. The second stage, at 380~500 °C, is due to the degradation of polyolefins formed in the first stage and the further dehydration of MH.

In comparison to EVA/MH, the initial degradation temperatures (T_5_) of EVA/MH/PCS, EVA/MH/PCS/DVB, and EVA/MH/PCS+DVB were all increased. In addition, both T_max1_ and T_max2_ rose more than 10 °C, indicating the thermal stability of the EVA composites was improved by PCS. Interestingly, there were few differences in the first weight loss stage among the three EVA materials containing PCS, but great differences were generated for EVA/MH/PCS/DVB in the second weight loss stage. Compared with EVA/MH/PCS, the T_max2_ of EVA/MH/PCS/DVB was 6 °C higher and the R_max2_ decreased by 55.5%, which illustrates the better effect of cross-linked PCS on the thermal stability of EVA composites.

Based on the thermal degradation parameters of the flame retardant (Table 4) and the zero residues of the EVA resin, the theoretical residues of the EVA composites were calculated. The theoretical residues of EVA/MH, EVA/MH/PCS, and EVA/MH/PCS/DVB were 35.0%, 35.9%, and 36.2%, which were consistent with the experimental values in Table 6, indicating that MH, MH/PCS, and MH/PCS/DVB have no catalytic charring effect on the EVA resin. It is worth noting that the residue of EVA/MH/PCS/DVB was a little higher than that of EVA/MH/PCS, indicating that the cross-linked PCS helped to increase the residual char of the EVA composites.

### 3.6. The Formation Mechanism of the Condensed Phase

**Figure 13:**
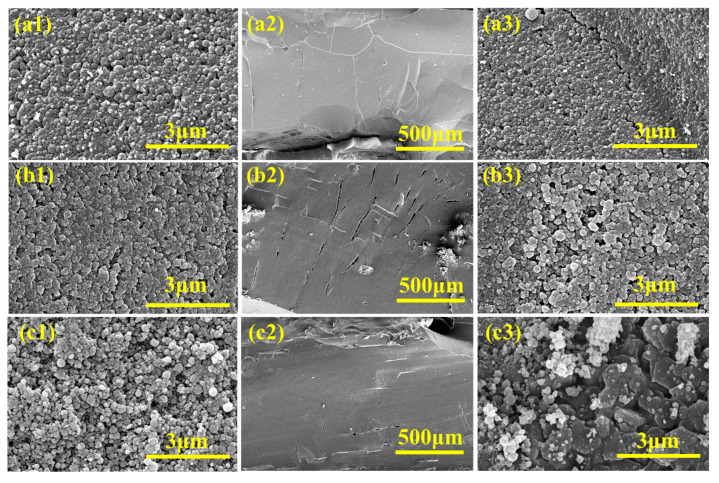
The microstructures of carbon residues after combustion of composites: (**a**) EVA/MH, (**b**) EVA/MH/PCS, and (**c**) EVA/MH/PCS/DVB. Image 1 is the surface, and images 2 and 3 are cross-sections.

The microstructures of the residual chars formed after the combustion of EVA/MH, EVA/MH/PCS, and EVA/MH/PCS/DVB are revealed in Figure 13. There are no differences in the surface char layers of the three composites. The surface char layers are composed of close-packed spherical particles (Figure 13(a1–c1)). The char structures in the cross-sections of the residues show differences. There are many long intersecting cracks in the residues of EVA/MH composites, while there are many vertical upward channels that are convenient for gas release and short parallel cracks in the residues of EVA/MH/PCS and EVA/MH/PCS/DVB (Figure 13(a2–c2)). Thanks to the existence of gas channels, the residual char can be free of violent shocks by the degradation gas, and the continuous surface char layer can be maintained. In addition, the particle shape in the cross-section of the residual char of EVA/MH/PCS/DVB maintains the original platelet shape of MH, which is different from that of EVA/MH and EVA/MH/PCS (Figure 13(a3–c3)), indicating that PCS/DVB covering the MH surface plays a non-negligible role in impeding the solid reaction between the molten EVA and MH.

**Figure 14:**
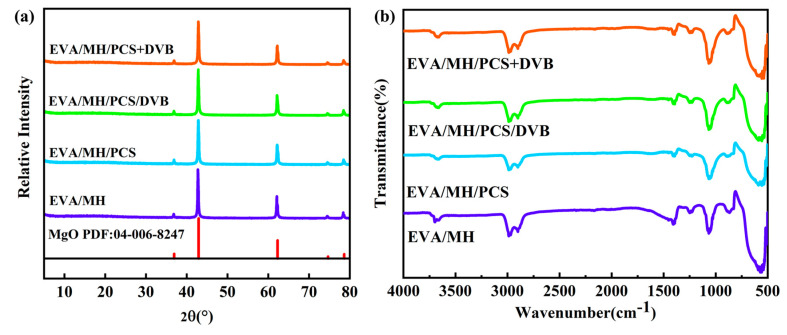
The XRD diffraction patterns (**a**) and the FTIR spectra of the residual char (**b**).

Figure 14 shows the XRD diffraction patterns and the FTIR spectra of the residual char of the composites after combustion. The residual char was composed of magnesium oxide, as evidenced in Figure 14 a. However, except for the absorption peaks of the Mg-O stretching vibration at 536 cm^−1^ in the FTIR curves, there was a stretching vibration of carbonates at 1431 cm^−1^ in the EVA/MH residue, as well as Si-O stretching vibrations at 1100 cm^−1^ in the residues of the rest of the EVA composites, which indicates that an amorphous inorganic-phase substance existed in the residues.

**Figure 15:**
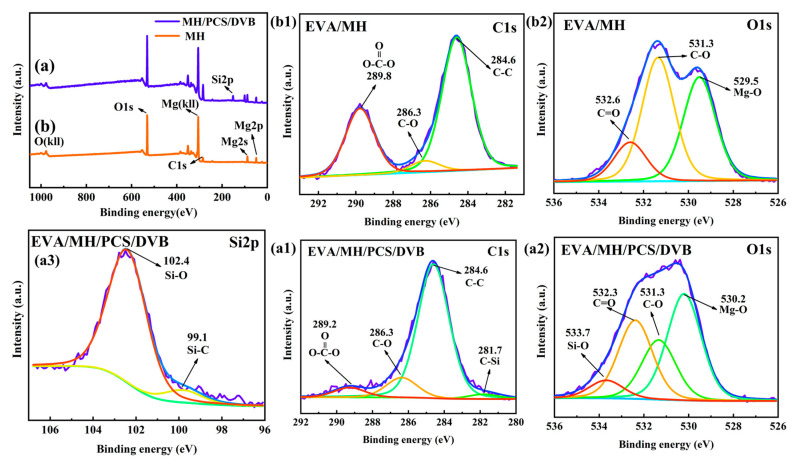
Total XPS spectra of residual char for EVA/MH and EVA/MH/PCS/DVB (**a**); high-resolution XPS spectra of C1s (**b1**) and O1s (**b2**) for residual char of EVA/MH; and C1s (**a1**), O1s, (**a2**), and Si2p (**a3**) for residual char of EVA/MH/PCS/DVB.

XPS was adopted to further reveal the binding states of the elements in the residues of EVA/MH and EVA/MH/PCS/DVB. The XPS spectra are given in Figure 15, and the contents of the elements are given in Table 7. For the residue of EVA/MH, the binding states of C1s (Figure 15(b1)) at 286.3 eV and 289.8 eV were attributed to C-O [40] and C=O [41], respectively. The binding states of O1s (Figure 15(b2)) at 531.3 eV and 532.6 eV were attributed to C-O and C=O, respectively, and were probably due to carbonate formed by the oxidation of generated coke and a reaction with magnesium oxide at a high temperature after the decarboxylation of EVA resin [39]. In addition, the element ratio of O to Mg was 2.5:1 (Table 7), far more than the 1:1 ratio in magnesium oxide, indicating the existence and oxidation of coke.

For the residue of EVA/MH/PCS/DVB, the binding states of C1s (Figure 15(a1)) at 281.7 eV and O1s (Figure 15(a2)) at 531.7 eV were attributed to C-Si and Si-O, respectively, which could be further confirmed by the Si-O (102.4 eV) and Si-C (99.8 eV) of the Si2p binding site (Figure 15(a3)). In addition, the area ratio of the Si-C (99.8 eV) to Si-O (102.4 eV) binding state was 9:100, lower than the 1:2 ratio shown in Figure 6, which indicates that the PCS was oxidized and improved the anti-oxidation of the residual char.

**Figure 16:**
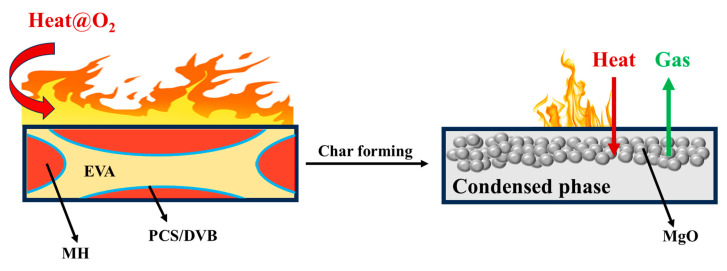
Schematic diagram of the char formation process of the EVA composite.

Based on the above analysis, the formation of the condensed phase of EVA/MH/PCS/DVB composites during combustion can be summed into two aspects, as shown in Figure 16. Firstly, coke formed after the oxidation of the removed ester group in the EVA resin, and magnesium carbonate formed on the surface of the magnesium oxide after the solid reaction between the oxidized coke and the magnesium oxide. The coke and magnesium carbonate improved the adhesive strength between magnesium oxide particles. Secondly, an amorphous complicated compound produced by the oxidation and degradation of PCS in the EVA/MH/PCS/DVB composites during combustion, which acted as a binder and anti-oxidant, further improved the strength and oxidation resistance of the condensed phase.

## 4. Conclusions

Based on this study of the flame-retardant properties and combustion behaviors of EVA/MH/PCS/DVB composites, the results show that the flame-retardant MH/PCS/DVB showed high flame-retardant efficiency in the LOI test and had a good effect on the control of smoke release, which was due to the improved strength of the residual char due to cross-linked PCS. In addition, the thermal stability and the residues of the MH and EVA/MH composites changed little due to PCS/DVB in comparison with PCS, which indicates that the improved flame retardancy of the EVA/MH/PCS/DVB composites lies in the condensed phase. The PCS cross-linked by DVB can leave more products in the residue during combustion and acts as a binder that can effectively join magnesium oxide particles together; thus, an intensive residual char with a special structure forms.

## Figures and Tables

**Table 1:** Compositions of the EVA composites.

Samples	EVA (wt.%)	MH (wt.%)	PCS (wt.%)	DVB (wt.%)
EVA/MH	50	50	0	0
EVA/MH/PCS	50	47	3	0
EVA/MH/PCS/DVB	50	47	2	1
EVA/MH/PCS+DVB	50	47	2	1

**Table 2:** The FTIR absorption peaks and vibration modes of PCS, DVB, and PCS/DVB.

Bond	Vibration Mode	Wave Number (cm^−1^)		
		PCS	DVB	PCS/DVB
C-H in benzene	Stretching	--	3087, 3058, 3006	3015
C-H in -CH_2_-	Stretching	2955, 2895	2966, 2930, 2873	2922
Si-H	Stretching	2100	--	2100
C=C in -CH=CH_2_	Stretching	--	1630	--
C=C in benzene	Skeleton vibration	--	1595, 1577, 1510,1480	1595,1500, 1445
C-H	Bending in plane	1410,1359	1400	1410, 1359
Si-CH_3_	Deformation	1250	--	1250
Si-O	Stretching	1020	--	1020
C-H in -CH=CH_2_	Bending out of plane	--	989	1000
C-H in benzene	Bending out of plane	--	906, 844, 800, 707	890, 709
Si-C in Si-CH_2_-Si	Stretching	830	--	830

**Table 3:** The contents of elements in MH and MH/PCS/DVB.

Element	MH (Atomic %)	MH/PCS/DVB (Atomic %)
Mg	22.78	7.76
O	59.03	40.98
C	18.20	39.99
Si		11.27

**Table 4:** TG and DTG parameters of flame retardants.

Samples	MH	MH/PCS	MH/PCS/DVB
T_5_ (°C)	369	389	389
T_10_ (°C)	383	399	402
T_max1_ (°C)	388	404	413
R_max1_ (°C/min)	−0.66	−0.91	−0.73
Residue_600°C_ (%)	70.0	71.9	72.4

**Table 5:** The combustion parameters of EVA composites.

Sample	EVA/MH 50/50	EVA/MH/PCS 50/47/3	EVA/MH/PCS/DVB 50/47/2/1	EVA/MH/PCS+DVB 50/47/2+1
TTI (s)	74	71	68	60
tpHRR_1_ (s)	280	201	219	195
pHRR_1_ (kW/m^2^)	438.8	291.1	396.7	450.6
tpHRR_2_ (s)	-	296	-	-
pHRR_2_ (kW/m^2^)	-	426.6	-	-
THR_6min_ (MJ/m^2^)	90	84	90	89
TSP_6min_ (m^2^)	5.2	7.3	5.4	6.9
Residue_6min_ (%)	36.9	38.7	40.5	39.5
FPI (s·m^2^/kW)	0.17	0.17	0.17	0.13
FGI (kW/m^2^·s)	1.24	1.16	1.38	1.77
THRI_6min_ (MJ/m^2^)	1.94	1.89	1.93	1.92
TSPI_6min_ (m^2^)	0.68	0.88	0.67	0.72

**Table 6:** TG and DTG parameters of the composites.

Sample	EVA/MH 50/50	EVA/MH/PCS 50/47/3	EVA/MH/PCS/DVB 50/47/2/1	EVA/MH/PCS+DVB 50/47/2+1
T_5_ (°C)	325	349	351	349
T_10_ (°C)	335	360	362	360
T_50_ (°C)	472	484	484	484
T_max1_ (°C)	336	365	369	366
T_max2_ (°C)	467	479	485	476
R_max1_ (°C/min)	−0.93	−0.74	−0.93	−0.95
R_max2_ (°C/min)	−1.96	−2.95	−1.31	−1.43
Residue_600s_ (%)	35.1	35.9	36.4	35.8

**Table 7:** Contents of elements in EVA/MH and EVA/MH/PCS/DVB in XPS testing.

Element	EVA/MH (Atomic %)	EVA/MH/PCS/DVB (Atomic %)
Mg	22.36	19.5
O	56.28	54.44
C	21.36	18.97
Si	-	7.09

## Data Availability

Data are contained within the article.
